# 基于临床路径进行的肺癌外科质量监测与持续改进：单一手术组经验总结

**DOI:** 10.3779/j.issn.1009-3419.2017.04.05

**Published:** 2017-04-20

**Authors:** 兴 王, 石 阎, 亚旗 王, 楠 吴

**Affiliations:** 100142 北京，北京大学肿瘤医院暨北京市肿瘤防治研究所胸外二科，恶性肿瘤发病机制及转化研究教育部重点实验室 Key Laboratory of Carcinogenesis and Translational Research (Ministry of Education), Department of Thoracic Surgery Ⅱ, Peking University Cancer Hospital & Institute, Beijing 100142, China

**Keywords:** 临床路径, 肺切除, 胸腔镜, Standard operation procedure, Lung resection, Video-assisted thoracoscopic surgery

## Abstract

**背景与目的:**

医疗流程标准化可提高治疗规范程度、控制合并症和住院时间，但国家制定的临床路径需根据各中心实际情况进行个体化调整。

**方法:**

本研究分析单一手术组进行肺切除手术患者的围手术期信息，通过对比术后平均住院日、医疗费用、手术胸腔镜占比等多个因素，从结构性指标、过程性指标、结果性指标三个方面进行了手术质量持续改进的总结和探索，寻找适合本手术组实际情况的理想术后住院时间及质量评估指标。

**结果:**

2016年的术后平均住院日较2013年明显缩短[(4.08±1.80) d *vs* (6.13±3.60) d, *P* < 0.001]，全胸腔镜手术占比（17%→48%→68%→73%）及单操作孔胸腔镜占比（0%→2%→52%→66%）四年来显著提高。

**结论:**

通过外科单元进行自我质量监控和改进，能够显著降低术后平均住院日，减少术后并发症发生。

## Abstract (en)

**Background and objective:**

Standard operation procedure (SOP) could standardize treatment procedure and reduce medical expenses, but SOP drawn by the government may not suit all medical institutions, so individual adjustment made by surgical team is necessary.

**Methods:**

We recorded the information of patients undergoing lung resection, including postoperative hospital stay, medical expenses and ratio of video-assisted thoracoscopic surgery (VATS) rate to evaluate surgical quality sustaining improvement in terms of structure indicator, process indicator and outcome indicator, aiming at exploring the ideal postoperative hospital stay and the best indicators to evaluate surgical quality improvement.

**Results:**

The average postoperative hospital stay reduced significantly between 2016 and 2013 [(4.08±1.8) d *vs* (6.13±3.6) d, *P* < 0.001)]. VATS rate increased from 2013-2016 (17%→48%→68%→73%), as well as single port VATS rate (0%→2%→52%→66%).

**Conclusion:**

Surgical quality surveillance and sustaining improvement by surgical team could further reduce postoperative hospital stay and perioperative complications.

临床路径目前在临床医学的各个领域内受到广泛应用，其目标是保证患者所接受的治疗项目标准化、精细化、程序化，减少治疗过程的随意化，因此可以提升各种疾病治疗的规范程度，提高医院资源的管理质量和利用率，加强临床治疗的风险控制，起到缩短患者住院天数、降低医疗费用的作用^[1]^。但国家临床路径规定相对宽泛，往往只能规定最低标准。如术前住院日规定≤7 d，术后恢复≤14 d^[2]^，如以此作为标准进行衡量，则使许多单位失去了持续改进的动力和标准。因此各外科单元，即由主任医师，主治/副主任医师，住院医师三级医师所组成的外科工作小组需要根据所在医院，科室等情况进行个体化的调整，优化临床路径，实现持续改进的目的。

手术质量管理是临床路径中重要的一个环节。目前有一些研究对肺癌的围手术期管理问题提出了基于循证医学的指标，根据其性质可大概分为结构性指标，过程性指标和结果性指标三类。

结构性指标用来评估医疗机构所提供服务的系统因素，如医院患者流量及是否具有专业胸外科资质为最关键指标；过程性指标用来评价患者在临床实践中常规采用某项特定操作的情况，如患者采取靶向治疗的比例，或支气管袖式肺叶切除术相对于全肺切除术的比例等都是重要的过程性指标；结局性指标指外科操作的治疗结果的评价，如并发症率、术后住院日、死亡率、生存率等^[3]^。采用循证医学的指标能够帮助医院管理者了解目前临床路径当中存在的问题，指导未来改进措施^[4]^。

而目前对于手术单元进行自身手术质量监控和管理的研究较少，如一些研究以科室为单位进行了关于平均住院日的归纳总结^[5]^，也有一些研究致力于淋巴结活检方式的改善或胸腔镜学习曲线的探索^[6, 7]^，因此本文旨在总结笔者2013年-2016年四年间在单一手术组内进行手术质量持续改进的过程和结果，分析和总结影响围手术期管理质量的因素，确定评价手术质量的因素，给手术医师所在单位临床路径的改进提出基于循证医学和统计学的依据，同时也使医师基于可量化的指标进行有效的学术交流。

## 1 资料与方法

### 1.1 数据来源及外科手术组组成

本研究对2013年-2016年由北京大学肿瘤医院胸部肿瘤外科二病区单一手术组患者的数据进行回顾性的分析。自制肺癌患者住院相关信息调查表以及使用Microsoft Excel应用程序创建肺癌患者住院相关信息调查数据库。数据库内容包括患者围手术期相关指标，并定期进行随访。根据数据内容，可划分为三类指标对医疗质量进行评估。结构性指标：评估医疗机构所提供服务的系统因素；过程性指标：评价患者在临床实践中常规采用某项特定操作的情况；结局性指标：评价外科操作的治疗结果。

### 1.2 临床路径的建立和改进

本组于2013年参考国家及医院标准拟定针对我组的肺手术路径，部分项目根据实际医疗空间进行相应调整，以便提高效率，如术前检查项目、抗生素使用、术后康复锻炼及拔管时间等。每年12月针对该年度数据进行分析和总结，并设定次年的质量改进目标（临床路径细则见<a href="http://www.lungca.org/files/2017-03-OA-0073-supplement.pdf">http://www.lungca.org/files/2017-03-OA-0073-supplement.pdf</a>）。

### 1.3 治疗模式

所有肺手术均符合手术适应症，部分复杂病例经过医院多学科协作组讨论后进入手术流程。手术入路主要以全腔镜手术为主，部分病例根据特殊情况采用腔镜辅助小切口或者开放手术切口方式进行。肺手术根据病变的不同部位，包括肺叶切除术、亚肺叶切除术、支气管袖式肺叶切除术（部分行血管成形手术）及全肺切除术等。术后患者根据病理分期给予标准的后续治疗或严密随访。

### 1.4 淋巴结清扫质量控制及并发症管理

淋巴结清扫方面，针对肺部恶性病变，影像学表现为实性结节，或者实性成分超过50%的磨玻璃病变，均施行系统性纵隔淋巴结清扫术及肺门和肺内各站淋巴结切除术。影像学表现为实性成分小于50%的磨玻璃病变，病理确诊后进行系统性纵隔淋巴结活检术及肺门和肺内淋巴结切除术。淋巴结切除过程中遇到细小管道给予钛夹夹闭。留置一根24 F胸管，个别患者术中漏气、引流较多或者复杂成型手术放置2根24 F胸管。标本离体后，由主治医师级别以上进行肺段及亚段淋巴结分检工作。

在术后过程管理方面，自2014年1月1日起，所有患者在术后第2日开始如无明确乳糜瘘或持续漏气，给予间断胸腔引流管夹闭，达到胸管拔除指征后移除胸管。

### 1.5 统计学方法

使用SPSS 20.0进行数据计算。根据数据正态分布与否，采用*t*检验或者*Wilcoxon rank* test来比较两组连续变量的差异，另外采用中位数来描述非正态分布数据。

## 2 结果

### 2.1 结构性指标

① 手术量：肺手术量在全年手术台数，病床数不变的情况下，维持逐渐递增的趋势，从2013年的年肺手术77台增至2014年107台、2015年的141台以及2016年的157台。②临床路径情况：2013年入路径比例为15.6%（12/77），出路径比例为0%，变异率为1.3%（1/77）；2014年入路径比例为91.6%（98/107），出路径比例为6.5%（7/107），变异率为1.9%（2/107）；2015年入路径比例为84.4%（119/141），出路径比例为7.1%（10/141），变异率为0.7%（1/141）；2016年入路径比例为87.9%（138/157），出路径比例为5.7%（9/157），变异率为0.6%（1/157）。其中主要出路径原因为良性病变，仅2015年1例患者因超过路径规定住院时间7 d为由出路径。

### 2.2 过程性指标

手术采用全胸腔镜占全年手术量比例如下：从2013年-2016年，手术采用全胸腔镜占全年手术量比例分别为：17%（13/77）、48%（51/107）、68%（96/141）及73%（114/157），呈逐渐递增趋势（图 1A）。

**1:**
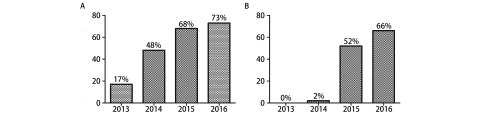
2013年-2016年胸腔镜手术及单孔胸腔镜手术占比。A：胸腔镜手术；B：单孔胸腔镜手术 Ratio of VATS surgery and single port VATS surgery from 2013 to 2016. A: VATS; B: single port VATS. VATS: video-assisted thoracoscopic surgery

单操作孔胸腔镜使用占全年手术量比例如下：2013年-2016年，手术采用单操作孔微创手术占全年手术量比例分别为：0%、2%（1/51）、52%（73/141）及66%（103/157），也呈逐渐递增趋势（图 1B）。

### 2.3 结局性指标（并发症率和术后住院日）

#### 2.3.1 质量改进第一阶段（2013年）并发症率和术后住院日的基本信息

术后并发症包括术后出现的轻型合并症例如一过性发热、皮下气肿、心律失常等，也包括较为严重的合并症例如需要气管镜下治疗的肺不张、需要二次手术的乳糜胸和出血、肺部感染、肺栓塞、声音嘶哑、持续漏气、和心脑血管意外等事件。统计发生例数如下，2013年度：18例（23%），中位术后住院日为5 d。详细分布如下。

术后住院日≤4 d的比例为34%，21%的患者术后住院日为5 d，23%的术后住院日为6 d，超过6 d的为22%。分析原因发现：2013年患者术后住院日≤5 d者未见并发症发生；术后住院日为6 d者为18例，2例为咳痰无力（11%），16例无特殊（89%）；术后住院日>6 d共17例，其中16例（94%）出现合并症，主要原因为乳糜胸、发热、感染等，1例（6%）无特殊原因。因此术后住院日超过5 d患者被规定为主要管控对象，同时住院日时间延长的主要原因为术后并发症。

#### 2.3.2 通过管控合并症和胸腔引流量在后续三个年度中（2014年-2016年）缩短术后住院日

以2013年全年数据为基础，设定4 d为目标术后住院日，严格管控超过5 d的住院比例，主要内容为减少合并症发生和管控胸腔引流量（包括术中应用钛夹及术后早期夹管等方法）。通过上述改进，2014年-2016年合并症情况如下：2014年：13例（12%），2015年：6例（4%），2016年：29例（18%）。

2014年主要并发症：漏气3例（23%）、咳痰无力3例（23%）、不明原因发热3例（23%）。

2015年主要并发症：发热、气胸、咳痰无力、下肢静脉血栓、体温高、肺栓塞各1例（16.7%）。

2016年主要并发症：不明原因发热5例（17.2%）、心律失常4例（13.8%）、肺不张、发热4例（13.8%），因术者亲自进行术后即刻记录，因此对于之前并未记载的术后呃逆、腹泻等并发症也有详细记录。

2014年术后住院日分布改进情况：术后住院日≤4 d的比例为61%，21%的患者术后住院日为5 d，7%的术后住院日为6 d，超过6 d的为10%。2015年术后住院日分布：术后住院日≤4 d的比例为66%，16%的患者术后住院日为5 d，11%的术后住院日为6 d，超过6 d的为8%。2016年术后住院日分布：术后住院日≤4 d的比例为71%，18%的患者术后住院日为5 d，5%的术后住院日为6 d，超过6 d的为5%。

2014年-2016年中位术后住院日降低为4 d，同时术后住院日>6 d的比例逐渐减低，平均住院日对比2016年与2013年有显著统计学差异[(4.08±1.80) d *vs* (6.13±3.60) d, *P* < 0.001]（图 2）。另外，术后超过14 d的病例数从2013年-2016年分别为：5例、0例、1例、0例。

**2:**
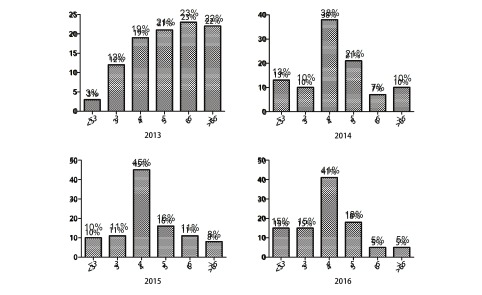
2013年-2016年术后住院日统计。2013年中位术后住院日5 d，2014年中位术后住院日4 d，2015年中位术后住院日4 d，2016年中位术后住院日4 d Mean postoperative hospital stay. 5 days (2013), 4 days (2014), 4 days (2015) and 4 days (2016)

考虑出入院手续及其他特殊原因，规定术后住院日>6 d为超预期术后住院日，超预期术后住院日比例逐渐降低；2013年为17例（22%），主要原因为乳糜瘘、发热、感染等；2014年为11例（10%），主要原因为漏气、不明原因发热；2015年为11例（8%），主要原因为“无特殊”；2016为11例（7%），主要原因为肺不张、肺栓塞等。

4年内共2例（2/482）死亡病例，1例为支气管袖式肺叶切除术后吻合口瘘合并出血导致术后死亡，另1例为肺癌术后疑似肺栓塞继发多器官脏器衰竭死亡。

#### 2.3.3 质量控制

非良性病变淋巴结清扫中位数分别为2013年16枚（0-39）、2014年16枚（0-40）、2015年17枚（0-46）、2016年15枚（0-35），根治性肺叶切除手术均符合NCCN制定的“肺门及纵隔淋巴结清扫各大于三站，总数大于6枚”标准。

#### 2.3.4 良性病变比例

笔者所在手术单元致力于降低肺切除良性病变的比例，自2013年-2016年起，切除肺占位为良性比例为12%（9/77）、7%（8/107）、10%（14/141）、10%（16/157）。

## 3 讨论

外科单一手术组通过自我管理进行手术质量持续改进是十分必要的。首先，国家所制定的肺癌诊疗临床路径具备普适性，但缺乏个性化设置，对于不同单位及不同级别医师的指导意义是不一致的。因此医师需根据自身单位及个人条件进行一定程度的调整，在不违反国家指定方针及原则的前提下，以质量改进为目标进行修订^[2]^。如笔者所在外科手术组以“减少术后住院日，降低并发症发生率”为主要目标进行持续改进，采用“戴明环”管理学方法有效地进行改进^[8]^，通过四年来的努力逐步实现了目标，提高了医疗质量，优化了临床路径在我单位的实施。

质量控制的重心在于自我监控和改进措施的实现。笔者单位在2012年就单病区肺癌外科淋巴结清扫工作进行了质控分析，提出肺癌外科手术质控应遵循严格的国际标准，以期提高分期诊断的准确性^[9]^。与此同时，我们进一步着眼围手术期患者管理，制定改进的目标。我们将降低术后平均住院日，降低并发症率作为主要目标，以期患者快速康复，提高床位使用率，降低次均费用，因此所做改进的措施均围绕此目标展开。如严格遵循临床路径进行操作，分析术后合并症的种类和频次，在接下来的时间段内有针对性的进行管控，减少主要合并症的发生。同时针对淋巴结清扫后胸腔引流等主要影响住院时间的因素进行研究总结，采用多种方法减少胸腔引流产生或者促进渗液回吸收，例如术中采用钛夹进行淋巴管结扎^[10]^、术后采用间断夹闭引流管等外科方法来降低术后引流量^[11]^，减少术后带管时间及住院时间等。

质量持续改进是针对每一段时间的数据进行回顾分析，并提出改进目标，经过一段时间的运行后再次进行总结。例如术后住院日方面，我们发现在2013年主要平均住院日较分散，而且术后住院时间较长。主要的原因在于拔管指征及并发症管控（例如持续漏气和乳糜瘘等）。根据文献中的提示我们调整了拔管流程，并针对引流进行一系列主动的外科管理措施，加快管道拔除。通过这些方法，我们发现从2014年开始，术后平均住院日集中在4 d。之后我们再进行2014年的总结和整理，发现虽然并发症率得到相应控制，但是患者对于术后住院日降低存在依从性较差问题，于是我们从医师层面加强患者对出院预期的合理引导，告知快速康复和功能锻炼的益处。于是发现2015年的术后4 d出院患者的比例进一步升高，2016年超预期住院的患者进一步减少。质量改进的原因可能在于，在并发症发生率得到有效控制的前提下，患者引流控制环节得到了加强，即医生主动加强患者快速康复的理念，从“医生要求患者快速康复”转变为“医患配合进行快速康复”的理念。

三级医师查房制度在质量改进中发挥着重要的作用。住院医师主要负责患者接诊，查体，术前检查、术前准备工作，另外作为术中助手并负责术后的常规处置等；主治医师主要负责术前患者准备工作的质量控制，术中助手及部分操作的主要执行者；主任医师负责术前患者访视，术中作为主刀医生，术后负责每日查房，并作为上级医师监管整个治疗流程。2016年起，除了住院医师正常的病案记录以外，术者还设计了专门的数据库在患者住院期间主动记录围手术期情况，特别是各种术后合并症的情况。我们的研究发现，在此改进措施实行之后，并发症率在数据呈现上升趋势，然而术后住院时间并没有延长，反而术后超过6 d住院日的情况更少，因而该结果并非反映并发症增加，而可能的原因是由于记录的更详细、及时，使得并发症统计更加全面。大部分单位目前仍采用后期回顾性查询病历的方法进行数据库建设，我们认为这样的方法虽然省力，但可能会受到病程记录准确性、真实性、全面性的影响，同时记录人员不同，也可能会存在偏移。通过前瞻性设计得当的数据记录或者采集对于胸外科临床数据收集有重要意义。另外，在我们的工作当中很重要的一点是鼓励任何级别的医生对治疗流程中不足的地方提出意见及建议，之后小组讨论决定改进方案后认真执行，并定期反馈改进结果。

同时临床路径也能够一定程度上配合研究工作的开展，在病例标准化的基础上较容易设计前瞻性的临床试验，取得满意的结果，不但如此，我们也可以采用可量化的指标进行胸外科医师间的有效交流，提高共同进步。

临床路径的变异分析和处理对临床路径的实施与不断完善具有重要意义。一方面，通过变异分析，医疗机构可以发现管理存在的问题，不断提高医院管理水平。然而我国与其他国家的变异率差别主要在于我国起步较晚，因此变异的涵义、鉴别与记录不健全。在我们的分析中也存在类似的问题，例如有些变异会无法识别，目前最大导致变异可能性的原因仍为“良性疾病”。仍需要科室及院内进一步的优化路径规范来提高系统的规范性^[12]^。

在过程性指标的探索当中，如胸腔镜的比例方面我们每年都有非常显著的进步，同时国内外目前有一系列研究探索胸腔镜肺叶切除术的学习曲线问题等^[7, 13]^，可供我们借鉴学习。同样笔者也认为，在丰富的开胸肺手术基础上培养胸腔镜技术是安全可行的方法。例如四年来单操作孔全腔镜手术的比例逐渐升高，而手术安全依然得到了充分的保证。另外笔者认为在指导年轻医师进行腔镜操作时，应首先选择叶裂发育较好、胸腔内无粘连、肿瘤较小的病变进行尝试，术者在扶镜手位置进行指导，如出现术中出血量较大，视野不清的情况应果断选择中转开胸^[14]^。

在外科手术组手术质量持续改进方面，我们也存在一些不足。客观因素方面，硬件系统不能配合临床需求实时采集数据，因此应通过完善健全的his系统将数据库内容与运行病历相关数据进行自动关联，协助医师进行更准确完善的数据采集。另外，主动进行自我分析是目前阻碍质量改进的最重要主观因素。由于劳动强度较大，需主诊医师专门辟出时间进行整理分析，并且需定期开展临床质量改进相关讨论，对团队的合作及凝聚力要求很高，不易长期坚持。但我们也认为，这本身对各级医师的临床技能和科研思维都是很好的锻炼，同时未来可设计相关指标来对外科手术组质量改进的情况进行系统性评价，并引进激励机制等。

因此我们认为，外科手术组自我进行手术质量持续改进，优化临床路径是一种较好实现并且获益很大的行为，可有效地提高医疗、教学及科研的质量，有较高的推广价值。
